# Glycan modifications to the gp120 immunogens used in the RV144 vaccine trial improve binding to broadly neutralizing antibodies

**DOI:** 10.1371/journal.pone.0196370

**Published:** 2018-04-24

**Authors:** Rachel C. Doran, Gwen P. Tatsuno, Sara M. O’Rourke, Bin Yu, David L. Alexander, Kathryn A. Mesa, Phillip W. Berman

**Affiliations:** 1 Department of Biomolecular Engineering, University of California, Santa Cruz, Santa Cruz, California, United States of America; 2 Department of Molecular, Cell and Developmental Biology, University of California, Santa Cruz, California, United States of America; 3 Gladstone Institute of Virology & Immunology, San Francisco, California, United States of America; Emory University School of Medicine, UNITED STATES

## Abstract

To date, the RV144 HIV vaccine trial has been the only study to show that immunization can confer protection from HIV infection. While encouraging, the modest 31.2% (P = 0.04) efficacy achieved in this study left significant room for improvement, and created an incentive to optimize the AIDSVAX B/E vaccine immunogens to increase the level of vaccine efficacy. Since the completion of the RV144 trial, our understanding of the antigenic structure of the HIV envelope protein, gp120, and of the specificity of broadly neutralizing monoclonal antibodies (bN-mAbs) that bind to it, has significantly improved. In particular, we have learned that multiple families of bN-mAbs require specific oligomannose glycans for binding. Both of the monomeric gp120 immunogens (MN- and A244-rgp120) in the AIDSVAX B/E vaccine used in the RV144 trial were enriched for glycans containing high levels of sialic acid, and lacked critical N-linked glycosylation sites required for binding by several families of bN-mAbs. The absence of these epitopes may have contributed to the low level of efficacy achieved in this study. In this report, we describe our efforts to improve the antigenic structure of the rgp120 immunogens used in the vaccine by optimizing glycan-dependent epitopes recognized by multiple bN-mAbs. Our results demonstrated that by shifting the location of one PNGS in A244-rgp120, and by adding two PNGS to MN-rgp120, in conjunction with the production of both proteins in a cell line that favors the incorporation of oligomannose glycans, we could significantly improve the binding by three major families of bN-mAbs. The immunogens described here represent a second generation of gp120-based vaccine immunogens that exhibit potential for use in RV144 follow-up studies.

## Introduction

The RV144 clinical trial has been the only human clinical trial to show that vaccination can provide protection from HIV infection [1]. The RV144 vaccination protocol consisted of immunization with the ALVAC (VCP1521) canarypox virus vector [2], designed to elicit a robust cell-mediated immune response, followed by co-immunization with the bivalent AIDSVAX B/E gp120 vaccine, designed to elicit an anti- gp120 antibody response [3–5]. This regimen provided statistically significant protection (Vaccine Efficacy = 31.2%, P = 0.04) over 3.5 years, with up to 60% efficacy within the first year after vaccination [1]. Follow-up analysis revealed that protection correlated with: antibodies to the V2 domain of gp120, high levels of antibody-dependent cellular cytotoxicity (ADCC) [6], and HIV-1 specific IgG3 antibodies [7], but not with gp120-specific CD8+ T-cell responses [1]. Together, these studies indicated a role for anti-gp120 antibodies in the modest but significant level of protection afforded by the vaccine. The importance of the antibody response was further supported by additional antibody binding studies [8, 9] and sieve analysis of breakthrough viruses [10]. Such studies associating protection with anti-gp120 antibodies provided a rationale for further development of gp120-based immunogens.

Since the completion of the RV144 trial, we have accumulated considerable insight regarding the structure of gp120, as well as of the specificity of neutralizing antibodies against it. The isolation of bN-mAbs from HIV-infected individuals revealed highly conserved protein and glyco-peptide epitopes on gp120 that were unknown when the AIDSVAX/BE vaccine was first developed. Of particular relevance was the identification of oligomannose terminal glycans targeted by multiple families of bN-mAbs. These glycans are located at conserved N-linked glycosylation sites in the V1/V2 domain (N301 and N332), near the apex of the gp120 trimer, and near the stem of the V3 domain [11–21], referred to as the “high mannose patch” [17]. The apparent preference of these bN-mAbs for gp120 within trimeric structures, as compared to monomeric gp120, suggested a requirement for quaternary structure for bN-mAb binding [18, 19]. However, it is becoming apparent that differences in glycan processing and glycan accessibility between monomeric and trimeric gp120 structures, in part, can account for this preference. While trimeric gp120, the functional unit of gp120 displayed on the surface of virions, is enriched for oligomannose glycans, recombinant monomeric gp120 displays predominantly complex, sialic acid-terminal, glycans [22, 23]. This discrepancy is at least partially explained by incomplete glycan processing in the ER and Golgi Apparatus, thought to be a consequence of steric hindrance to glycosidase enzymes during trimer formation [21, 24].

The AIDSVAXB/E immunogens were produced in a Chinese Hamster Ovary (CHO) cell line, and consequently possessed a high degree of N-linked glycan sialylation [25]. High sialic acid content is desirable for a majority of biotherapeutics, as its presence in recombinant glycoproteins is known to impart a longer in vivo half-life [26, 27]. However, it is now apparent that sialic-acid moieties on gp120 occlude critical bN-mAb epitopes [25, 28, 29]. Although previously unappreciated in HIV vaccine design, N-linked glycosylation is now recognized as a significant determinant of the antigenic structure of HIV envelope glycoproteins [11, 13]. Here we describe our efforts to improve the antigenic structure of the vaccine immunogens used in the RV144 clinical trial by altering the location of no more than two critical N-linked glycosylation sites and constraining the types of glycoforms incorporated. These simple changes improved the antigenic structure of gp120 immunogens used in the RV144 trial, as measured by improved binding by three major families of bN-mAbs. We desired to minimize the number of modifications to the RV144 gp120 immunogens necessary to enhance the presentation of conserved glycan epitopes that are displayed on virions and recognized by bN-mAbs. While correlates of protection in the RV144 clinical trial indicate the importance of anti-V1V2 antibodies, it is unclear exactly which structural features of the gp120 immunogens were important in eliciting the observed protection, and whether or not these features can be faithfully recapitulated with gp120-fragment or trimeric immunogens. We propose that incrementally improving gp120 immunogens, while preserving as much of the observed (and perhaps unrecognized) structural elements of the gp120 immunogens therefore presents a logical approach in HIV vaccine design. The glycan-optimized gp120 proteins we describe represent second-generation vaccine immunogens that possess epitopes recognized by multiple bN-mAbs not present on the vaccine immunogens used in the RV144 trial. By enhancing the potential to elicit bN-mAbs associated with protection from HIV infection in passive transfer studies [30–32], the immunogens can be further investigated in follow-up immunization schedules to improve the efficacy of the RV144 vaccine regimen from 31.2% to a level of 50% or greater thought to justify clinical deployment [33].

## Materials and methods

### Production and purification of rgp120 glycan variants

Site-directed mutagenesis was used to create the MN- and A244-rgp120 glycosylation site mutants using a Gibson Assembly master mix (New England Biolabs, Ipswich, MA). The MN_GNE_ sequence differs from the MN sequence published by Gurgo et al. [34] by 18 amino acids within the gp120 sequence, and contains a 27 amino acid N-terminal purification tag from herpes simplex virus glycoprotein-D (gD). The A244_GNE_ sequence from the RV144 trial is unaltered from the original sequence described by McCutchan et al. [35] except for the addition of the N-terminal gD tag [4]. The mature recombinant A244-gp120 (A244-rgp120) protein contains the same primary amino acid sequence as the A244-rgp120 incorporated into the AIDSVAXB/E vaccine. The MN_GNE_ and A244 _GNE_ sequences have been described in detail and submitted to Genbank under the accession numbers MG189370 and MG189369, respectively. Nucleotide and protein alignments were performed with Geneious software (version 6.0; http://www.geneious.com, Kearse et al., 2012) [36], and all sequences and notations employ HXB2 numbering. Plasmids containing rgp120 were transiently transfected in either 293 GnTI^-^ HEK (ATCC CRL-3022) cells, deficient in the enzyme N-acetylglucosaminyltransferase I (GnTI^-^ 293 cells), or CHO-S cells (Invitrogen, Carlsbad, CA) using electroporation (MaxCyte STX, Gaithersburg, MD). All rgp120s contained an 11 amino acid N-terminal deletion, replaced by the N-terminal gD tag that was used for affinity chromatography protein purification as previously described [25]. Proteins were analyzed for molecular mass with SDS PAGE on 4–12% Bis-tris gel (Life Technologies, Carlsbad, CA). Purified, CHO-derived MN-rgp120 (MN_GNE_), was obtained from GSID (GSID, South San Francisco, CA).

### Physical characterization of gp120 proteins

Immunoprecipitations using Dynabeads™ Protein G magnetic beads (Life Technologies, Carlsbad, CA) were performed on MN gp120 glycan variants preceding Endo H digest. Briefly, 300μL of supernatant from gp120 transient transfections were incubated with 2.5μg purified mouse monoclonal antibody (34.1), raised against the N-terminal gD tag, to form rgp120/mAb immune complexes. Beads were re-suspended and incubated in transfection supernatant containing rgp120/34.1 immune complexes. All incubations were performed for one hour on a rotating platform, at room temperature. The beads were washed three times in PBST with a final wash in PBS, and used directly in glycosidase digests. Endo H digest was purchased from New England Biolabs (Ipswich, MA) and used to digest GnTI^-^ and CHO expressed rgp120 proteins according to the manufacturer’s instructions. Briefly, ~10μg of recombinant protein was denatured and reduced with 10X denaturation buffer, then boiled at 100°C for 10 min. Samples were incubated with 10X G5 reaction buffer and 5,000 units Endo H for 12h at 37°C. Digested and mock-digested samples were analyzed by polyacrylamide gel electrophoresis (PAGE) using precast polyacrylamide gels (4–12% Bis-Tris) in MOPS running buffer (NuPAGE^®^, Invitrogen, Carlsbad, CA). Blots were probed with the 34.1 mAb and visualized with a goat-anti-mouse HRP conjugated polyclonal (American Qualex Antibodies, San Clemente, CA).

### Immunoassays

A Fluorescence Immunoassay (FIA) was used to measure antibody binding to gp120. A FIA, which replaces the more common enzymatically catalyzed chemiluminescent readout (ELISA) with fluorescently labeled secondary polyclonal, is slightly less sensitive, but provides the benefit of a higher dynamic range of sensitivity in addition to higher reproducibility [37]. Briefly, 2μg/mL of an anti-gD mouse monoclonal antibody (mAb 34.1) was diluted into PBS and incubated overnight in 96 well black microtiter plates (Greiner, Bio-One, USA). Plates were blocked in PBS containing 1% BSA+0.05% normal goat serum in 0.01% thimerosal for two hours. Wells were incubated with either 6μg/mL of purified rgp120, or 25–200μL of rgp120 containing growth conditioned cell culture supernatant, overnight at 4°C. Three-fold serial dilutions of bN-mAb or polyclonal control sera were added starting at 10μg/mL, followed by incubation with a 1:3,000 dilution of goat-anti-human AlexaFluor 488 conjugated polyclonal antibody (Jackson ImmunoResearch Laboratories, West Grove, PA, Life Technologies, Carlsbad, CA). Dilutions were performed in a 1% BSA solution in PBS containing 0.01% thimerosal, and all incubations were performed for 90 min at room temperature followed by a 4Xwash in PBST buffer unless otherwise noted. Cell culture supernatants were normalized to contain approximately 2μg/mL of rgp120 for screening assays, or 4μg/mL for binding to the assays to an extended bN-mAb panel, and captured with 1μg/mL (for screening assays), or 2μ/mL of immobilized anti-gD 34.1 monoclonal antibody. Broadly neutralizing antibody binding curves were performed in quadruplicate for statistical confidence. Human IgGK was used as a negative control, protein-G purified rabbit polyclonal sera raised against rgp120 (PB94), was used as a coating control. Absorbance was read using an EnVision Multilabel Plate Reader (PerkinElmer, Inc, Waltham, MA) using a FITC 353 emission filter and FITC 485 excitation filter. The PG9 mAb was purchased from Polymun Scientific (Klosterneuburg, Austria), and the CH01-CH03 antibodies were gracious gifts of Dr. Barton Haynes at Duke University (Durham, NC). The following reagents were obtained through the NIH AIDS Reagent Program, Division of AIDS, NIAID, NIH: PGT126, PGT128, PGT 121, VRC01, and 10–1074, or were a gift from Dennis Burton (La Jolla, CA).

### Statistical analysis

Statistical analyses were performed with GraphPad Prism software (v5.0). A Kruskall-Wallis test with a Dunn’s post-hoc test, to correct for multiple comparisons, was used to determine statistically significant differences in EC50 (p<0.05) measurements of bN-mAb binding between CHO-derived, RV144 based immunogens and GnTI^-^ expressed gp120 glycan variants. Error bars represent the standard error of the mean (SEM).

## Results

### Improvement of the antigenic structure of A244-rgp120

**Fig 1:**
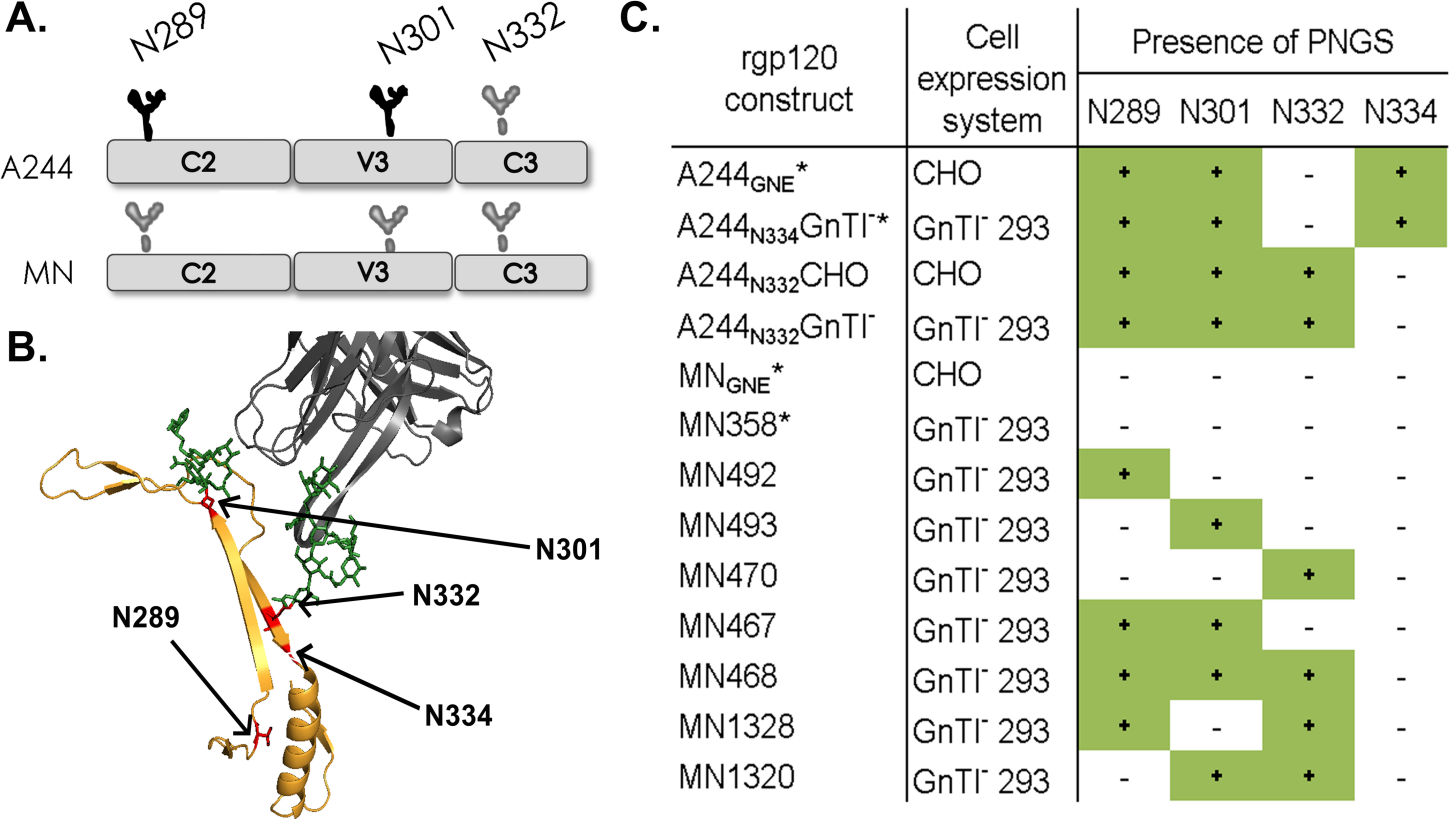
Modification of N-linked glycosylation sites in MN- and A244-rgp120. (**A**) The A244-rgp120 or MN-rgp120 sequences were analyzed for the presence of highly conserved glycans known to be important for bN-mAb binding within the C2-C3 domains. Glycosylation sites are represented as either black (present in RV144 immunogen) or grey (absent in original RV144 immunogen) structures. (**B**) A ribbon diagram depicts the 3-dimensional arrangement of the N289, N301, N332, and N334 PNGS. The structure is based on crystal structure of the BG505 SOSIP.664 gp140 trimer (in gold) bound to the PGT122 bN-mAb (in grey) [41]. The N301 and N332 glycan structures immobilized by the PGT122 antibody are indicated in green, while the asparagine residues at the base of relevant PNGS are indicated in red. (**C**) Site directed mutagenesis was used to create MN- or A244-rgp120 variants introducing one or more of the indicated PNGS. A summary of the PNGS variant constructs assayed is shown. The constructs with identical number and location of PNGS to the RV144 rgp120 immunogens are marked with an asterisk.

We analyzed the A244-rgp120 primary sequence for presence of bN-mAb associated glycans. Glycans can obscure even geographically distant epitopes [38, 39]. We therefore aimed to introduce a minimal number of additional potential N-linked glycosylation sites (PNGS), and limited our investigation to highly conserved (~70% across clade) [40] glycans incorporated into a minimal V3 stem bookended by the N289 and N334 PNGS (**Fig 1A and 1B**). Analysis of the A244-rgp120 sequence revealed that it lacked a PNGS located at HXB2 position N332, containing instead a glycan at position N334 that is highly typical amongst clade AE viruses. Based on these observations, we modified the A244-rgp120 sequence to contain point mutations E332N and N334S. These mutations resulted in the ablation of the N334 PNGS and introduction of a PNGS at N332. As the A244-rgp120 sequence contained all other highly conserved PNGSs within the considered range, no other glycan variants were investigated (**Fig 1C**).

**Fig 2:**
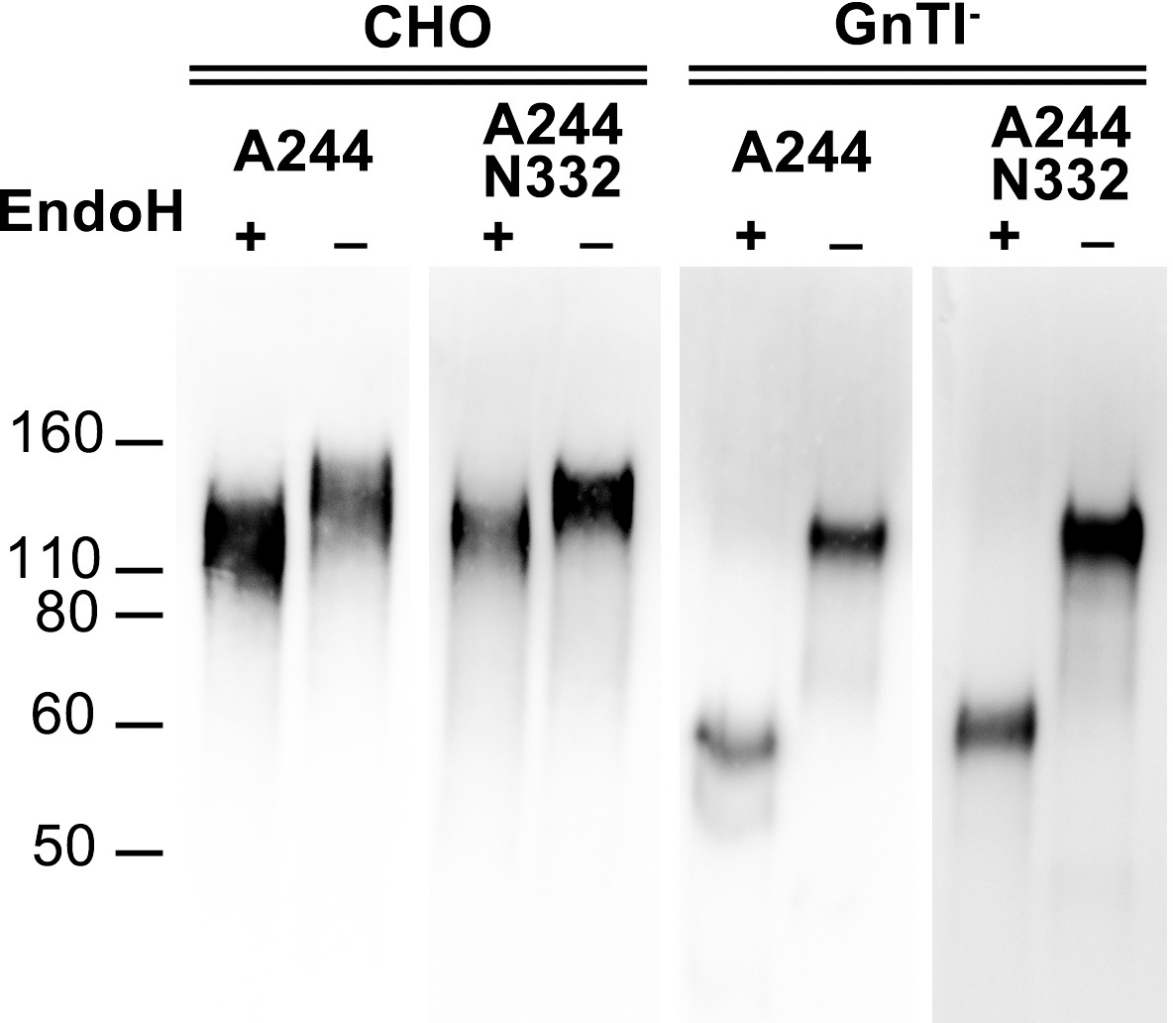
Endo H digest and immunoblot of A244 gp120 glycan variants. A244-rgp120s containing either N332 or N334 based PNGS were expressed in either CHO-S (lanes 1–4) or HEK 293 GnTI^-^ cells (lanes 5–8) via transient transfection. Purified protein was subjected to Endo H or mock digest (digest buffer alone), and analyzed for mobility on 4–12% reducing SDS-PAGE gels. Immunoblots were probed with the mouse monoclonal 34.1 that binds a conformation independent epitope in the N-terminal gD tag of all expressed proteins, and visualized with goat-anti-mouse HRP conjugated polyclonal sera.

Because multiple bN-mAbs bind mannose-5 or mannose-9 glycan epitopes, and such epitopes are destroyed when sialic acid terminal glycans are incorporated, we expressed the gp120 variants in GnTI^-^ 293 cells that limit N-linked glycosylation to predominantly mannose-5 terminal moieties [25]. To verify that the proteins produced in GnTI^-^ cells lacked complex sialic acid containing glycans, gp120 variants were treated with Endoglycosidase H (Endo H). Endo H is an enzyme that cleaves high mannose, but not complex, sialic acid containing N-linked oligosaccharides. Endo H treatment results in cleavage of uniquely oligomannose terminating N-linked glycosylation moieties, leaving behind only the base N-acetyl glucosamine residue from the original diacetylchitobiose core of the original glycan base. When digested with Endo H and run on an SDS page gel, the GnTI^-^ derived A244 gp120 glycan variants migrated as a ~60kD band, the predicted molecular weight of non-glycosylated gp120, while the highly-sialyted gp120 produced in normal CHO cells, like that used in the RV144 trial, remained mostly resistant to Endo H digestion (**Fig 2**).

**Fig 3:**
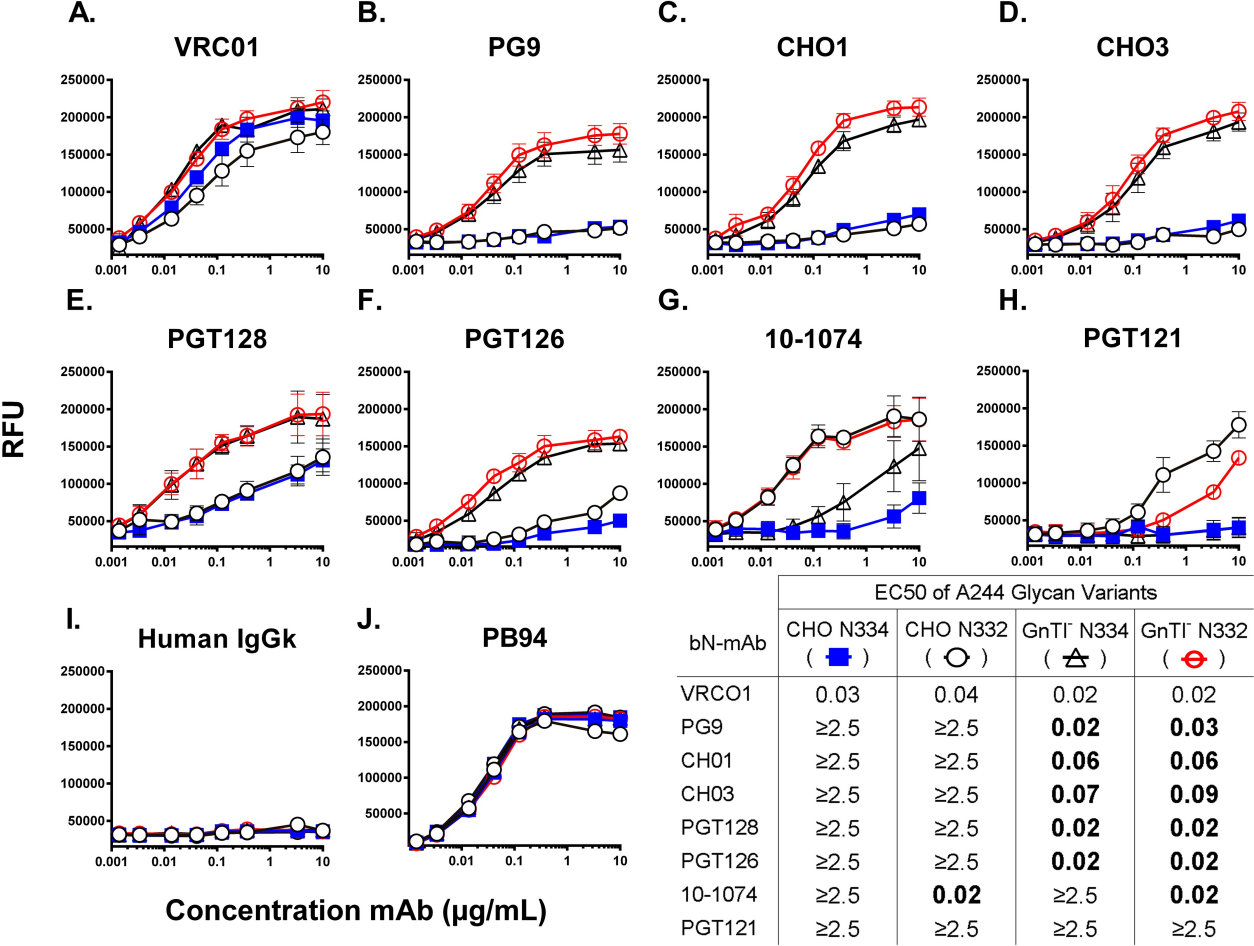
Binding of A244-rgp120 glycan variants to bN-mAbs. Purified A244-rgp120 glycan variants were compared by FIA for binding to a panel of bN-mAbs. Results are reported as the half maximal effective concentration (EC50) in μg/mL, or the concentration of antibody required for a half-maximal binding, measured in Relative Fluorescence Units (RFU) on a titration-binding curve. Values are reported as ≥2.5μg/mL if titration curves did not plateau, or mean EC50 was calculated as ≥2.5μg/mL. Each curve represents the average of four independent assays. The EC50 values of the rgp120 glycan variants that are significantly different (p<0.05) from the A244_GNE_ EC50 to the same bN-mAb are highlighted in bold. Human IgGK polyclonal antibody was used as a negative control, and binding curves to purified rabbit polyclonal antibody (PB94) raised against rgp120 were used as a coating control.

Previously, we have reported that rgp120s exhibit higher affinity to PG9, the prototypic V1V2 glycan dependent bN-mAb, when expressed in GnTI^-^ 293 as compared to CHO cells [25]. We expressed A244-rgp120 N332 glycosylation site variants in both CHO and GnTI^-^ 293 cells. The resulting proteins (A244_N332_CHO and A244_N332_GnTI^-^) were purified via affinity chromatography (see Materials and Methods) to investigate how the global change in glycoform processing would affect binding to a panel of bN-mAbs. Recombinant gp120 based on the A244-rgp120 sequence from the RV144 trial, containing the N334 PNGS, was expressed in CHO and GnTI^-^ 293 cells. The resulting proteins (A244_GNE_ and A244_N334_GnTI^-^, respectively) were assayed by FIA to identify how position of the PNGS or the type of glycosylation affected bN-mAb binding. EC50s derived from antibody binding curves were used to quantitate improvements to presentation of epitopes recognized by bN-mAbs. The binding curves and derived EC50s are summarized in **Fig 3**.

To verify that rgp120 glycan variants maintained relevant secondary and tertiary structure, we assayed gp120 binding to the bN-mAb VRCO1 that recognizes a conformation dependent protein epitope at the CD4 binding site of gp120 [42]. Both N332 and N334 GnTI^-^ expressed gp120 variants exhibited slight, though not statistically significant, improvements to the VRC01 bN-mAb as compared to CHO expressed rgp120 (**Fig 3A**). The PG9 bN-mAb that recognizes a mannose-5 dependent epitope in the V2 domain exhibited negligible binding to A244_GNE_, but bound with high affinity to both GnTI^-^ expressed N332 and N334 variants (EC50 of 0.02 and 0.03 μg/mL, respectively) (**Fig 3B**) While PG9 binding appeared dependent on the mannose-5 glycans resulting from GnTI^-^ expression, it did not distinguish between the N332 and N334 variants [43]. A similar binding preference was observed with the CH01 and CH03 antibodies that recognize a glycan-dependent epitope in the V2 domain [20].

The PGT128 and PGT126 antibodies belong to a family of bN-mAbs whose epitope lies at the base of the V3 domain and requires oligomannose glycans at N301 and N332 [18, 19, 44]. Both the N332 and N334 A244-rgp120 variants exhibited weak binding to PGT128 and PGT126 when produced in CHO cells. However, expression in GnTI^-^ 293 cells resulted in significant improvements in EC50 for both the A244 N332 and N334 variants. Both the A244_N332_GNTI^-^ and A244_N334_GnTI^-^ displayed binding curves with EC50s of 0.01μg/mL for PGT128 and 0.02μg/mL to PGT126 (**Fig 3E and Fig 3F**). While the bN-mAbs PGT121 and 10–1074 bN-mAbs have been reported to accommodate an N334 in place of an N332-based PNGS in a strain-dependent manner [14, 17], we found that both the A244 and MN-rgp120s required the N332 glycosylation site for optimal presentation of the PGT121 and/or 10–1074 bN-mAb epitopes. A244-rgp120 binding appeared to be strictly dependent on the presence of the N332 glycan. The A244_N332_ exhibited high affinity binding to 10–1074 (EC50 ~0.02μg/mL) regardless of cell expression system (**Fig 3G**). However, PGT121 exhibited marginally better binding to the CHO produced A244-rgp120, in concordance with the observation that PGT121 exhibits a preference for complex glycans (**Fig 3H**) [14]. Other members of the PGT family of bN-mAbs (PGT 130–145) exhibit absolute dependence on quaternary structure afforded by the trimeric form of gp120, and were therefore not included in our analysis [19]. These studies indicate that the antigenic structure of A244-rgp120, as measured by the binding of bN-mAbs from the PG9, PGT128, and PGT121 families, can be improved by replacement of complex glycans with oligomannose glycans and incorporation of the N332 PNGS.

### Improvement of the antigenic structure of MN-rgp120

Sequence analysis of MN-rgp120 revealed that the V3 stem lacked conserved PNGS at positions 289, 301, and 332. To evaluate the effects of these PNGS on the MN-rgp120 antigenic structure, we designed a series of glycosylation variants with single, double, or triple PNGS mutations. The first series of glycosylation mutants (MN492, MN493, and MN470) added a single PNGS at positions 289, 301, or 332, respectively. MN-rgp120 variants that incorporated two additional PNGS included: MN467 (two additional PNGS at N289 and N301), MN1320 (two additional PNGS at N301 and N332), and MN1328 (two additional PNGS at N289 and N332). Finally, the MN468 mutant introduced three additional PNGS at 289, 301, and 332. These rgp120 variants are summarized in **Fig 1C**. The MN-rgp120 gene encoding the same sequence of MN-rgp120 used in the RV144 trial was included as a comparator for bN-mAb binding studies. MN glycan mutant constructs were expressed in GnTI^-^ 293 and CHO cells via transient transfection. When expressed in CHO cells, the MN-rgp120 variants exhibited extensive proteolysis, rendering CHO-expressed protein unsuitable for assay. This proteolysis is consistent with previous observations that clade B gp120 sequences are particularly susceptible to digestion by secreted cellular proteases that clip at the GPGR motif within the tip of the V3 crown [45, 46]. Due to this extensive proteolysis, we were unable to produce sufficient, un-cleaved CHO-MNrgp120. However, we were able to obtain the highly purified CHO expressed MN-rgp120 immunogen (MN_GNE_) that was incorporated into the AIDSVAXB/E vaccine of the RV144 trial, to use for comparison.

**Fig 4:**
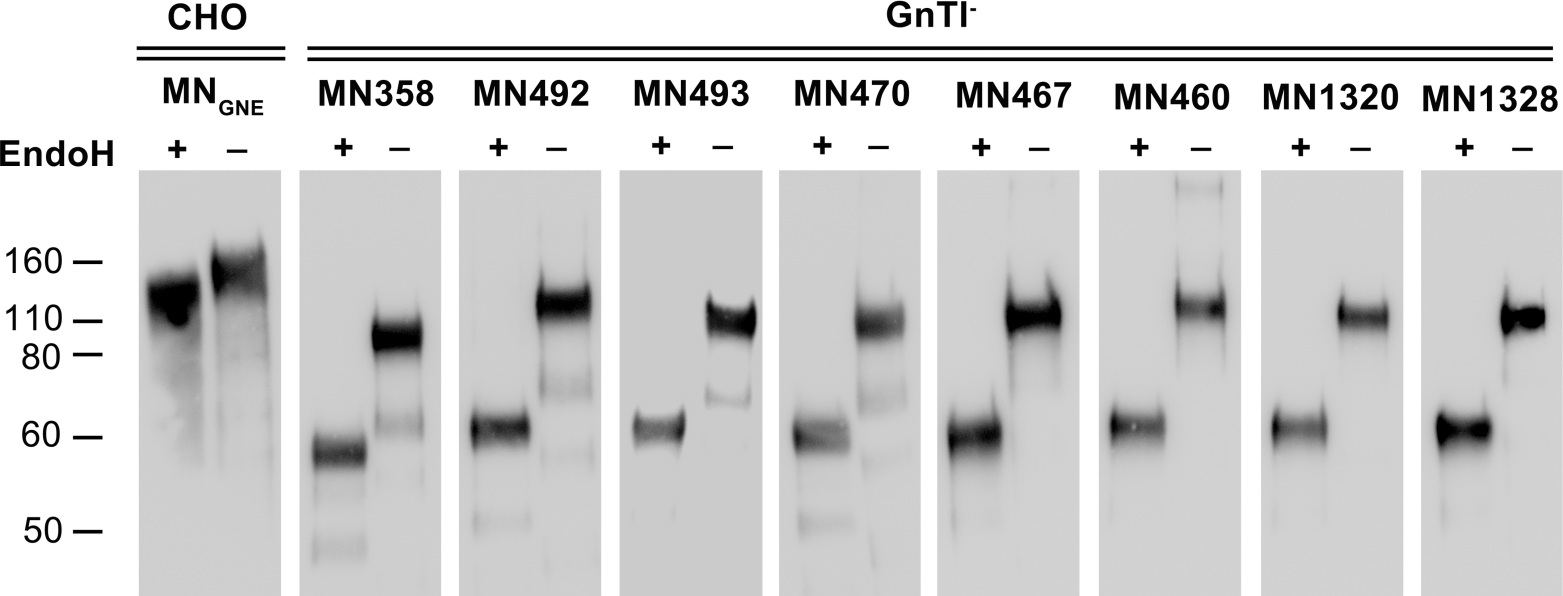
Endo H digest and immunoblot of MN gp120 glycan variants. Wildtype MN rgp120 expressed in CHO GnTI^-^ cells, or MN glycoform mutants with single, double, or triple glycan additions were expressed in GnTI^-^ cells via transient transfection. Purified MN_GNE_ or GnTI^-^ cells supernatants containing expressed rgp120 were immunoprecipitated via a monoclonal antibody to an N-terminal gD tag bound to protein-G coated beads. Immunoprecipiated rgp120 variants were subjected to Endo H or mock digest (digest buffer alone), and analyzed for mobility on 4–12% reducing SDS-PAGE gels. Immunoblots were probed with the mouse monoclonal 34.1 and visualized with goat-anti-mouse HRP-conjugated polyclonal sera.

Cell culture supernatants from transiently transfected GnTI^-^ cells expressing MN rgp120 with glycan epitope insertions were normalized for gp120 concentration and subjected to Endo H glycosidase digests to confirm size and oligomannose content (**Fig 4**). All mock-digested rgp120 GnTI^-^ expressed glycan variants ran as ~110kDa bands. Some MN rgp120 glycan mutants (UCSC 358, 470, 492, and 493) showed a minor amount of proteolysis which resulted in the appearance of a faint ~70kDa (undigested gp120) or ~50kDa (Endo H digested gp120) band on the immunoblots (**Fig 4**).

**Fig 5:**
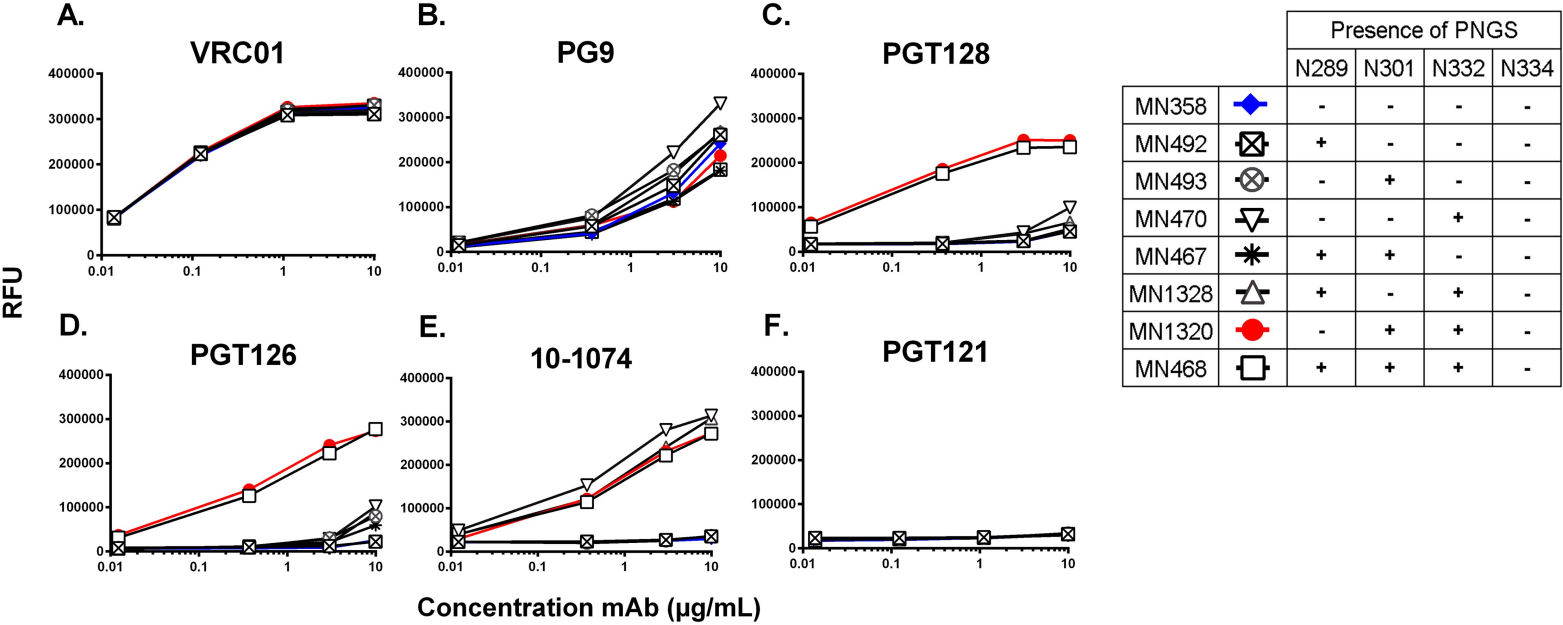
Screen of MN glycan mutant supernatants for improvements to bN-mAb binding. A FIA was used to identify 293 GnTI^-^ expressed MN-rgp120 glycan variants exhibiting improved bN-mAb binding profiles as compared to the wildtype MN sequence expressed in GnTI^-^ (MN358). Recombinant gp120s were expressed in GnTI^-^ 293 cells via transient transfection, and transfection supernatants were normalized to contain ~2μg/mL. MN-rgp120 variants were captured onto 96 well black plates using a 1μg/mL concentration of mouse monoclonal antibody to an N-terminal gD tag. Binding curves to the VRCO1 bN-mAb, which binds a conformation dependent epitope in the CD4 binding site, were used to assay for maintenance of overall secondary and tertiary structure. All screening assays were performed in duplicate. MN-rgp120 glycan variants were assayed for improved antigenicity to a panel of glycan dependent bN-mAbs to be considered for further analysis.

We used a FIA to screen the panel of eight GnTI^-^ expressed MN gp120 glycan variants for binding to bN-mAbs, and the EC50s derived from these results are summarized in **Fig 5**. Binding to the CD4 binding bN-mAb VRCO1 was unaffected by the addition of PNGS. While the bN-mAbs PGT128 and PGT126 required the addition of both the N301 and N332 PNGS, binding to MN constructs by the10-1074 bN-mAb was conferred by the minimal addition of the N332 PNGS (**Fig 5**). The addition of the N289 PNGS appeared to have no effect on binding of any bN-mAbs tested.

**Fig 6:**
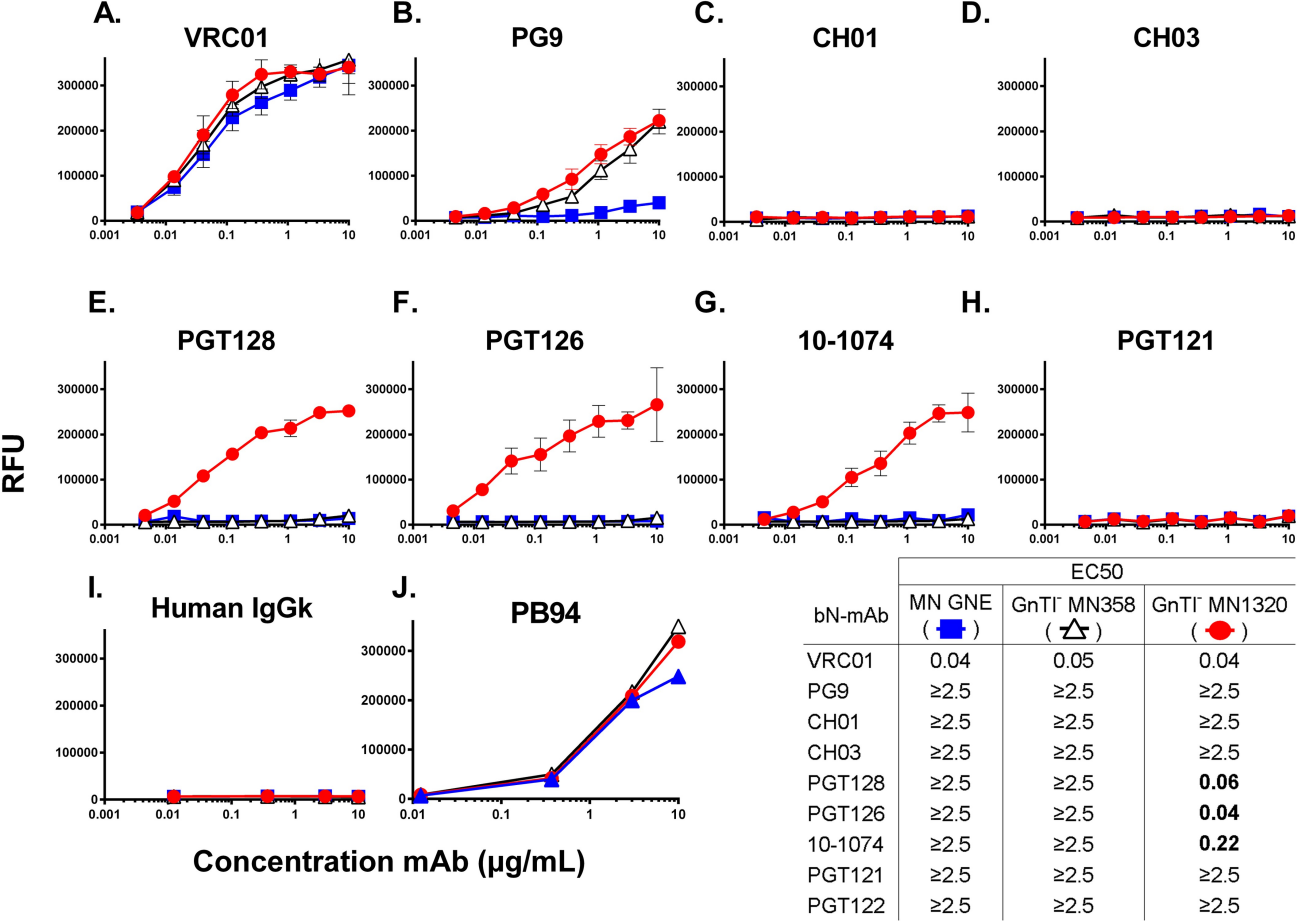
Binding of MN glycan variants to extended panel of bN-mAbs. The MN-rgp120 variants MN358 and MN1320 expressed in GnTI^-^ cells were compared to MN_GNE_ for improved binding to an array of bN-mAbs. Recombinant gp120s were expressed in GnTI^-^ 293 cells via transient transfection. Purified MN_GNE_ or transfection supernatants were normalized to contain 4μg/mL rgp120 and captured using 2μg/mL of mouse monoclonal antibody 34.1, then assayed by FIA for bN-mAb binding. Results are reported in μg/mL as (EC50), the concentration of antibody required for a half-maximal binding, measured in Relative Fluorescence Units (RFU) on a titration-binding curve. Values are reported as ≥2.5μg/mL if titration curves did not plateau or if mean EC50 was ≥2.5μg/mL. Binding curves to bN-mAbs were performed in quadruplicate. The rp120 constructs exhibiting statistically significant differences in EC50 values (p<0.05) from the MN_GNE_ are noted in bold. Human IgGK polyclonal antibody was used as a negative control and purified goat polyclonal antibody raised against rgp120 (PB94) was used as a coating control.

From the original panel of seven MN-rgp120 PNGS mutants assayed, we identified MN1320 GnTI^-^ as the construct containing the fewest PNGS additions (at N301 and N332) to confer improved bN-mAb antigenicity. We investigated the binding the MN1320 glycan variant to an extended panel of bN-mAbs (**Fig 6)**. For comparison, we included MN_GNE_, as well as its GnTI^-^ 293 produced cognate, MN358. Binding curves of the VRC01 bN-mAb were not statistically different amongst any of the MN-rgp120 glycan variants, regardless of additional PNGS or cell expression system (**Fig 6A**). In agreement with previous observations [43], the epitope defined by the PG9 bN-mAb was poorly represented on the MN_GNE_, but could be improved with incorporation of oligomannose glycoforms. However, expression of the MN358 and MN1320 in GnTI^-^ 293 cells was able to introduce weak binding. The PG9 epitope was not significantly affected by the insertion of PNGS in the V3 stem. Insertion of PNGS at N301 and N332 were necessary to introduce high binding to the V3 stem glycan binding bN-mAbs 10–1074, PGT126, and PGT128. However, CH01 and CH03 and PGT121 bN-mAbs exhibited no binding to any of the MN-rgp120 variants (**Fig 6**).

## Discussion

Following the RV144 correlates of protection analysis, various strategies have been pursued to improve the level of protective immunity observed (reviewed in Stephenson et al.) [47, 48]. Ongoing clinical trials have been designed to investigate variations on the RV144 protocol, including the use of envelope proteins from different clades or more robust viral vectors [48]. Other studies have been designed to maintain the original AIDSVAX/BE gp120 immunogens but alter such variables as number of booster injections, interval between booster injections, or risk-factor of the clinical trial population. However, little effort has been allocated to optimize the original rgp120 immunogens used in the trial. In this paper we use insights gained since the conclusion of the RV144 trial to improve the A244 and MN-rgp120 immunogens of the AIDSVAX B/E vaccine. The improvement of an existing vaccine with a modest record of efficacy offers several advantages. First, this approach builds upon a gp120 vaccine formulation with a demonstrated record of safety. Second, it utilizes existing manufacturing and production knowledge used to create similar molecules for commercial scale. Finally, the incremental improvement of a vaccine efficacy from the 31.2% observed in RV144 to the level of 50% or more thought to be required for product registration [33] presents a less formidable task than the development of an entirely new vaccine concept.

We found that we could significantly improve the antigenic structure of the AIDSVAXB/E immunogens by the addition of no more than two glycosylation sites, and by the modification of glycoform incorporated. The PG9, CH01, and CH03 bN-mAbs are members of a major class of neutralizing antibodies that binds a glycan-dependent epitope within the V1V2 domain. While A244_GNE_ displayed negligible binding to PG9, CH01, and CH03, the GnTI^-^ derived A244gp120 constructs (containing both N332 or N334 PNGS) exhibited drastic improvements in antigenicity to these antibodies. The dependence of GnTI^-^ expression for the A244_N332_ and A244_N334_ gp120s for binding to CH01, CH03, and PG9 antibodies supports a dependence of these antibodies on the mannose-5 glycan epitopes, as opposed to elements associated exclusively with quaternary structure [43, 49]. Additionally, the glycan optimized MN construct (GnTI^-^ expressed MN1320) exhibited improved binding to only the PG9 bN-mAb in a manner dependent on glycoform but not additional PNGS. However, MN1320 did not bind CH01 and CH03, regardless of cell expression system. This is likely a consequence of absent amino acid, rather than glycan epitope determinants, that were not covered in the scope of this study.

Both the MN and A244 glycan-optimized constructs additionally displayed statistically significant improved binding to the V3 glycan binding bN-mAbs PGT128 and PGT126 as compared to their respective RV144 cognates. Surprisingly, GnTI^-^ expression of both A244- and MN-rgp120 was found to enhance binding to the PGT126 and PGT128 bN-mAbs for both the N332 or N334 PNGS variants. While these bN-mAbs have been reported to recognize a mannose-5 glycoform at N301, the N332 glycoform recognized by the PGT128 family is characteristically mannose-8 or mannose-9 [12]. One potential explanation for improved binding profile of GnTI^-^ produced antigens to these bN-mAbs is that the accessibility of mannose-8/9 epitope at N332 is maintained, if not enhanced, in the context of the neighboring mannose-5 terminal glycans of GnTI^-^ 293 expressed gp120 proteins. These data indicate that production of gp120 immunogens in GnTI^-^ 293 cells can improve antigenicity not only to mannose-5 binding bN-mAbs, but also to mannose-8 or -9 dependent bN-mAbs [50, 51]. The CD4 binding site, recognized by the VRC01 bN-mAb, is a conserved, glycan-independent epitope. Although the VRC01 antibody has not been documented to directly contact a glycan residue [52], we observed a non-statistically significant, but consistent improvement in binding of VRCO1 to the MN and A244 gp120 proteins when presenting exclusively oligomannose glycoforms. This improvement, similar to that observed previously [28] was not dependent on the addition of any PNGS, and may, like improvement observed for the epitopes bound by the PGT128 family of bN-mAbs, be a result of improved protein epitope accessibility in the context of smaller, less charged glycans. The 10–1074 and PGT121 bN-mAbs displayed a requirement for an N332 based PNGS. This preference was glycoform independent; binding to A244_N332_ could not be improved with GnTI^-^ expression. Of note, PGT121 exhibited better binding to the CHO expressed A244_N332_ as compared to GnTI^-^ expressed A244_N332_; likely due to the preference of PGT121 for complex glycoforms [14].

Since the completion of the RV144 trial, at least five major sites of virus vulnerability on the HIV envelope protein have been reported. Three of these sites within gp120 are defined by the VRC01, PG9, and PGT128 epitopes. Outside of gp120, bN-mAbs target epitopes within the MPER domain of gp41 and at the interface between gp120 and gp41 subunits [19, 53–56]. Recent passive transfer studies with bN-mAbs indicate that a successful bN-mAb eliciting vaccine should raise at least two of these bN-mAb families to contend with expected viral escape mutants [30, 31, 57]. Different variations of envelope-protein based immunogens such as trimeric gp140s, gp120s, or scaffolded fragments, have as of yet not been able to consistently elicit broadly neutralizing antibodies to any of these sites [58–60]. Additionally, it has not been established how modifications to the RV144 protocol, via glycan optimization, trimerization of gp120, or use of different germline-targeting immunogens will affect the immunogenicity of the non-neutralizing V1V2-binding antibodies correlated with protection in the RV144 trial [1]. In this regard small modifications to the gp120 backbone such as those described here may be preferable to avoid major changes in antigenic structure associated with gp140 trimers or scaffolds that may disrupt presentation of critical V1V2 epitopes. It is possible that presence of oligomannose glycoforms on gp120 immunogens could potentially reduce immunogenicity, as the sialic residues of complex glycans are important in extending immunogen half-life in vivo [61, 62]. Additionally, terminal mannose glycan moieties on gp120 may act to repress gp120 immunogenicity by down-regulating the dendritic cell response [63], or by action of mannose receptors[61]. Conversely, the increased presence of mannose glycans could be argued to improve immunogenicity, via transport of mannose receptors to cross-presentation pathways [62] or the lack of sialic residues that contribute to suppression of B cell responses via CD22 (as a mechanism to limit self-recognition) [64]. However, studies investigating the role of glycoform on protein immunogenicity have reached conflicting conclusions [28, 43, 63, 65, 66]. The lack of consensus amongst the studies on the effect of glycoform on protein immunogenicity indicates that ultimately, downstream optimization of factors that modulate vaccine immunogenicity, such as formulation and adjuvants, may play a particularly important role. Further immunogenicity studies will be required provide insight to these questions.

As the AIDSVAX B/E, in conjunction with the VCP1521 canarypox vector, established a baseline of vaccine efficacy, building upon this immunization protocol offers a logical approach to optimizing a safe and effective vaccine. We propose that evaluating the efficacy of the immunogens described herein represents a systematic, stepwise modification in structure to improve vaccine efficacy. Various promising pathways exist for investigating modifications on a gp120-based vaccine protocol, including strategies that use a DNA prime-gp120 boost [67], more potent vector primes [48], or germline gene targeting strategies [50]. The immunogens using the glycan optimization strategies outlined in this report can be further investigated for potential use in follow up studies in conjunction, or in parallel with RV144 follow-up studies investigating the role of A244, MN, or other gp120 immunogens in the elicitation of a protective immune response.
